# Optimization of chamfer masks using Farey sequences and kernel dimensionality

**DOI:** 10.1038/s41598-022-11807-3

**Published:** 2022-05-10

**Authors:** Baraka Jacob Maiseli

**Affiliations:** Department of Electronics & Telecommunications Engineering, College of Information & Communication Technologies, University of Dar es Salaam, 14113 Dar es Salaam, Tanzania

**Keywords:** Engineering, Electrical and electronic engineering, Computer science, Mathematics and computing, Applied mathematics

## Abstract

Farey sequences have captured the attention of several researchers because of their wide applications in polygonal approximation, generation of Ford circles, and shape analysis. In this work, we extend the applications of these sequences to optimize chamfer masks for computation of distance maps in images. Compared with previous methods, the proposed method can more effectively generate optimal weights from larger chamfer masks without considering multiple and rather complex defining variables of the masks. Furthermore, our work demonstrates the relationship between size of the chamfer kernel, Farey sequence, and optimal weights of the chamfer mask. This interesting relationship, which may be useful in various image processing and computer vision tasks, has never been revealed by any other previous study. Results from the current research may advance our understanding on the applications of Farey sequences in computational geometry and vision-related tasks. To allow reproducibility of the results, implementation codes and datasets can be accessed in the public repository at https://www.mathworks.com/matlabcentral/fileexchange/71652-optimization-of-chamfer-masks.

## Introduction

Distance function (or metric) refers to the shortest path between pairs of points in a set. The concept forms an integral part in various engineering and science disciplines: in computer vision and image processing, for instance, distances can be used to compute the similarity of objects, extract skeletons from objects, predict path of a robot, and locate electronic components on the printed circuit board^1,2^; in weather forecasting, prediction models can be compared and evaluated using distance metrics^3^; and, in radar communications and imaging, one can locate the position of unknown object behind the building’s wall^4–6^. These applications, among several others in the literature, make studying and understanding metrics important.

One fascinating application of metrics involves the generation of distance maps from binary images. This (non-reversible) process can be achieved through a distance transform (DT)^7–9^, which maps contents of a binary image into positive grayscale values that show the degree of separation between objects and non-object features of an image. For a set of points, *P*, and for real-valued points *a* and *b*, the distance transform reduces to evaluating

1 $$\begin{aligned} \text {DT}(P)[a]=\min _{b\in P}\text {dist}(a,b), \end{aligned}$$

where $\text {dist}(\cdot )$ denotes the distance function: Euclidean^10^, Quasi-Euclidean, Chessboard, and Manhattan, among others. The $\text {dist}(\cdot )$ operator can be executed globally or locally over an image. Computation of DTs through local distance operations are preferred in most digital image processing tasks because such (neighborhood-driven) operations possess regularity properties, and hence can easily be implemented in processors. This research focuses on these neighborhood-driven operations, specifically those based on chamfer masks because they have attracted a wide interest of scholars^11–14^.

Chamfer masks can be designed using cost functions, which provide conditions to achieve optimum coefficient values of the masks. Errors associated with chamfer masks are usually computed with respect to a reference disc generated by the Euclidean metric. In Ref.^14^, the authors proposed a robust cost function, called RLog, which can be used to optimize chamfer masks. RLog gives an intuitive interpretation of the error between estimated and actual distances. But Maiseli et al. give rather daunting formulations to compute optimum chamfer values using RLog. In this work, we have discovered that similar results can be obtained through either Farey sequences^15,16^ or dimensionality (size) of the chamfer masks.

Based on the RLog cost function, this research gives closed-form simple equations that can be used to optimize chamfer masks using knowledge gained from patterns of the Farey sequences and from structure of the chamfer masks. Unlike the method by Maiseli et al., our approach allows easier computation of even high-order chamfer masks without involving multiple variables that define such masks. For instance, the proposed approach can generate optimal chamfer coefficients of $501 \times 501$ mask or even of higher orders without undertaking complicated derivations.

## Methods

### The RLog cost function

**Figure 1:**
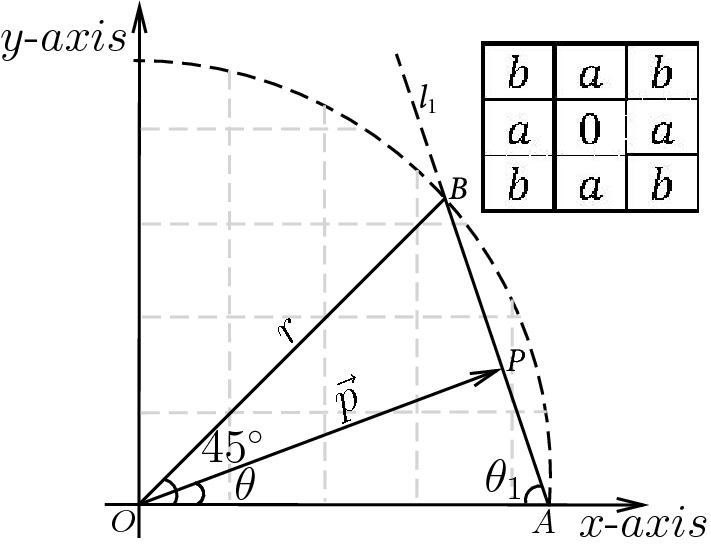
Octant portion of an eight-sided polygon generated by $3\times 3$ chamfer mask: $l_1, (b-a)y+ax=r$, $A(\frac{r}{a},0)$, $B(\frac{r}{b},\frac{r}{b})$, and $w(\theta )=|\vec {p}|$.

The RLog formula can comprehensively be understood by considering Fig. 1^14^, which shows portion of an eight-sided regular polygon created by a $3 \times 3$ chamfer mask. From the Figure, and according to the authors in Ref.^14^, RLog refers to logarithm of the relative accuracy between actual and estimated discs. The metric is defined by

2 $$\begin{aligned} Z(\theta )=\log \left( \frac{1}{r}w(\theta )\right) , \end{aligned}$$

where $r=\sqrt{x^2+y^2}$ denotes the actual disc radius, measured from the center, *O*(0, 0), to the point (*x*, *y*) on the disc’s circumference; $0\le \theta \le 45^{\circ }$ denotes the angle between the horizontal and a vector $\vec {p}$ from *O* to the chamfer edge ${\overline{AB}}$; and, $w(\theta )$ defines the length of $\vec {p}$.

Application of sine rule to $\bigtriangleup OAP$ in Fig. 1 yields

3 $$\begin{aligned} w(\theta )=\frac{r}{a\cos \theta + (b-a)\sin \theta }. \end{aligned}$$

Plugging (3) into (2) yields

4 $$\begin{aligned} Z(\theta )=-\log (a\cos \theta + (b-a)\sin \theta ), \end{aligned}$$

which defines the variation of RLog with an angle $\theta$.

Considering arbitrary shapes of chamfer masks, and focusing on the edge of the chamfer polygon corresponding on the maximum RLog,  (4) can be generalized to

5 $$\begin{aligned} Z(\theta )=-\log (a\cos \theta + (\psi -t) a\sin \theta ), \end{aligned}$$

with $\psi$ and *t* dependent on shape of the chamfer mask^14^ (Table 1). Analyzing the trend of $\psi$ in Table 1, we found that $t=\lfloor \psi \rfloor$.

**Table 1:** Maximum RLog under unoptimized and optimized conditions of the Chamfer masks.

Chamfer mask	$\psi$	$t=\lfloor \psi \rfloor$	$(a_{\text {opt}},\beta _{\text {opt}})$	Unoptimized RLog	Optimized RLog ($\%$)
$3\times 3$	$\sqrt{2}$	1	(0.9612,1.3593)	3.4385	1.7192
$5\times 5$	$\sqrt{5}$	2	(0.9865,2.2060)	1.1776	0.5888
$7\times 7$	$\sqrt{10}$	3	(0.9935, 3.1418)	0.5644	0.2822
$9\times 9$	$\sqrt{17}$	4	(0.9962, 4.1076)	0.3266	0.1633
$11\times 11$	$\sqrt{26}$	5	(0.9976, 5.0866)	0.2119	0.1059
$13\times 13$	$\sqrt{37}$	6	(0.9983, 6.0724)	0.1482	0.0741
$15\times 15$	$\sqrt{50}$	7	(0.9987, 7.0622)	0.1094	0.0547
$17\times 17$	$\sqrt{65}$	8	(0.9990, 8.0545)	0.0840	0.0420
$19\times 19$	$\sqrt{82}$	9	(0.9992, 9.0485)	0.0665	0.0333
$21\times 21$	$\sqrt{101}$	10	(0.9994, 10.0436)	0.0539	0.0270
$23\times 23$	$\sqrt{122}$	11	(0.9995, 11.0397)	0.0446	0.0223

The graph of $Z(\theta )$, with coefficients of $\cos \theta$ and $\sin \theta$ unoptimized, depicts major and minor error lobe(s) generated by a chamfer mask (Fig. 3). Our central goal is to minimize the maximum $Z(\theta )$ located on a major error lobe. This goal can be achieved by considering^14,17^

6 $$\begin{aligned} Z(0^{\circ })=\max _{\theta }\left\{ |Z(\theta )|\right\} . \end{aligned}$$

The right side of (6) evaluates to a maximum RLog of

7 $$\begin{aligned} Z_{\text {m}}=\log \left( a_{\text {opt}}\sqrt{(\psi -\lfloor \psi \rfloor )^2+1}\right) , \end{aligned}$$

located at an angle $\theta _{\text {m}}=\arctan (\psi -\lfloor \psi \rfloor )$, where $a_{\text {opt}}$ denotes an optimal chamfer weight that generates the lowest possible RLog. Combining (6) and (7) gives

8 $$\begin{aligned} a_{\text {opt}}=\frac{1}{\root 4 \of {(\psi -\lfloor \psi \rfloor )^2+1}}. \end{aligned}$$

The other optimized chamfer weight controlling the major error lobe is $\beta _{\text {opt}}=\psi a_{\text {opt}}$. Plugging (8) into (7) gives

9 $$\begin{aligned} Z_{\text {m}}=\frac{1}{4}\log ((\psi -\lfloor \psi \rfloor )^2+1), \end{aligned}$$

which depends only on $\psi$. This equation is relatively simpler, unlike those from classical approaches that demand knowledge on multiple chamfer weights to compute maximum errors generated by chamfer masks.

In the following section, we show that Farey sequences and sizes of chamfer masks can further simplify the process of optimizing chamfer masks. The derived equations and relationships have never been explored by previous studies, and may be employed to simplify optimizations of high-order chamfer masks.

### Optimization of chamfer masks using Farey sequences

Given an integer, $n\ge 1$, then the Farey sequence, $F_n$, contains a set of irreducible fractions $\frac{p}{q}$, where $0\le p\le q\le n$ and $(p,q)=1$, arranged in ascending order. Following this rule, the first five Farey sequences become

$$\begin{aligned} F_1&=\left\{ \frac{0}{1},\frac{1}{1}\right\} ,\\ F_2&=\left\{ \frac{0}{1},\frac{1}{2},\frac{1}{1}\right\} ,\\ F_3&=\left\{ \frac{0}{1},\frac{1}{3},\frac{1}{2},\frac{2}{3},\frac{1}{1}\right\} ,\\ F_4&=\left\{ \frac{0}{1},\frac{1}{4},\frac{1}{3},\frac{1}{2},\frac{2}{3},\frac{3}{4},\frac{1}{1}\right\} ,~~\text {and}\\ F_5&=\left\{ \frac{0}{1},\frac{1}{5},\frac{1}{4},\frac{1}{3},\frac{2}{5},\frac{1}{2},\frac{3}{5},\frac{2}{3},\frac{3}{4},\frac{4}{5},\frac{1}{1}\right\} . \end{aligned}$$

Inspecting fractions of the Farey sequence from $n \ge 2$, one can observe an odd number of terms for each sequence, with the middle term being $\frac{1}{2}$. In addition, successive terms of the Farey sequence share a common property: if $\frac{p_1}{q_1}$, $\frac{p_2}{q_2}$, and $\frac{p_3}{q_3}$ represent three successive Farey sequences, then (http://mathworld.wolfram.com/FareySequence.html)

10 $$\begin{aligned} q_1p_2-q_2p_1=1, \end{aligned}$$

and

11 $$\begin{aligned} \frac{p_2}{q_2}=\frac{p_1+p_3}{q_1+q_3}. \end{aligned}$$

The authors in Ref.^18^ stated that (10) and (11) are equivalent, and can be useful in determining a middle term sandwiched between neighboring terms in the Farey series. For example, computing $\frac{p_2}{q_2}$ requires inserting $\frac{p_1+p_3}{q_1+q_3}$, called the mediant fraction, between $\frac{p_1}{q_1}$ and $\frac{p_3}{q_3}$^18,19^.

Consider (8) and the Farey sequence, $F_n=\{\frac{p_1}{q_1}, \frac{p_2}{q_2},\frac{p_3}{q_3}\ldots \}$. Through inspection and analysis, we found that $\psi$ in (8) relates with $q_2$: $\psi =\sqrt{{q_2}^2+1}$ and $\lfloor \psi \rfloor =q_2$. Using these findings, (8) becomes

12 $$\begin{aligned} a_{\text {opt}}=\frac{1}{\root 4 \of {\left( \sqrt{{q_2}^2+1}-q_2\right) ^2+1}}, \end{aligned}$$

implying that $\beta _{\text {opt}}=(\sqrt{{q_2}^2+1})a_{\text {opt}}$. Furthermore, investigating patterns of $F_n$, we discovered that $q_2=n$ (denominator of the second term from the left side of the sequence) for all values of $n>0$. Therefore, (8) reduces to

13 $$\begin{aligned} a_{\text {opt}}=\frac{1}{\root 4 \of {\left( \sqrt{{n}^2+1}-n\right) ^2+1}}, \end{aligned}$$

and $\beta _{\text {opt}}=(\sqrt{{n}^2+1})a_{\text {opt}}$. Following a similar approach, the maximum RLog can be computed as

14 $$\begin{aligned} Z_{\text {m}}=\frac{1}{4}\log \bigg (\bigg (\sqrt{n^2+1}-n\bigg )^2+1\bigg ). \end{aligned}$$

Farey sequences have traditionally been used to address some problems in Mathematics: generation of Ford circles, approximation of irrational numbers, establishment of Fibonacci numbers, and explanation of the Riemann hypothesis. In this work, we have demonstrated that Farey sequences may as well be used to optimize chamfer masks, and to compute maximum errors associated with polygons generated by such masks. This application has, despite its high demand in vision-related tasks, never been thoroughly explored by previous scholars.

Furthermore, we discovered an interesting relationship between Farey sequences and shapes of the chamfer masks: Let $\Omega$ be size of the chamfer mask, then $n=\lfloor \frac{\Omega }{2}\rfloor ~\forall n$. Plugging this relationship into (13) and (14) yields

15 $$\begin{aligned} a_{\text {opt}}=\frac{1}{\root 4 \of {\left( \sqrt{{\left\lfloor \frac{\Omega }{2}\right\rfloor }^2+1}-\left\lfloor \frac{\Omega }{2}\right\rfloor \right) ^2+1}}, \end{aligned}$$

where $$\beta _{\text {opt}}=\left( \sqrt{{\left\lfloor \frac{\Omega }{2}\right\rfloor }^2+1}\right) a_{\text {opt}}$$, and

16 $$\begin{aligned} Z_{\text {m}}=\frac{1}{4}\log \bigg (\bigg (\sqrt{\left\lfloor \frac{\Omega }{2}\right\rfloor ^2+1}-\left\lfloor \frac{\Omega }{2}\right\rfloor \bigg )^2+1\bigg ). \end{aligned}$$

In this case, the value of $Z_{\text {m}}$ occurs at $\theta _{\text {m}}=\arctan \left( \sqrt{\left\lfloor \frac{\Omega }{2}\right\rfloor ^2+1}-\left\lfloor \frac{\Omega }{2}\right\rfloor \right)$. Equations (15) and (16), where $a_{\text {opt}}$ and $\beta _{\text {opt}}$ solely depend on $\Omega$, simplifies the task of optimizing chamfer masks. We can, therefore, compute optimal weights of high-order chamfer masks without considering multiple defining variables—an advantage that increases convenience in designing the masks (Table 2, Fig. 4). Note that, based on (15) and (16), the locations of the forward–backward masks on the binary image can be intuitively determined as

17 $$\begin{aligned} {M = \left\lfloor \frac{\Omega + 1}{2}\right\rfloor ^2 + \left\lfloor \frac{\Omega }{2} \right\rfloor ^2}. \end{aligned}$$

Table 2 shows comparable results of the optimal chamfer weights computed by our method and that of Maiseli et al.^14^. The implication of this comparability is that the derived formulas for optimizing chamfer masks generate accurate results.

**Table 2:** Comparisons of optimized chamfer masks.

Chamfer mask	*n*	$(a_{\text {opt}},\beta _{\text {opt}})$ ^14^	$(a_{\text {opt}}^{(n)},\beta _{\text {opt}}^{(n)})$	$(a_{\text {opt}}^{(\Omega )},\beta _{\text {opt}}^{(\Omega )})$	RLog ($\%$) ^14^	$\hbox {RLog}^{(n)}$ ($\%$)	$\hbox {RLog}^{(\Omega )}$ ($\%$)
$3 \times 3$	1	(0.9612, 1.3593)	(0.9612, 1.3593)	(0.9612, 1.3593)	1.7192	1.7192	1.7192
$5 \times 5$	2	(0.9865, 2.2060)	(0.9865, 2.2060)	(0.9865, 2.2060)	0.5888	0.5888	0.5888
$7 \times 7$	3	(0.9935, 3.1418)	(0.9935, 3.1418)	(0.9935, 3.1418)	0.2822	0.2822	0.2822
$9 \times 9$	4	(0.9962, 4.1076)	(0.9962, 4.1076)	(0.9962, 4.1076)	0.1633	0.1633	0.1633
$11\times 11$	5	(0.9976, 5.0866)	(0.9976, 5.0866)	(0.9976, 5.0866)	0.1059	0.1059	0.1059
$13\times 13$	6	(0.9983, 6.0724)	(0.9983, 6.0724)	(0.9983, 6.0724)	0.0741	0.0741	0.0741
$15 \times 15$	7	(0.9987, 7.0622)	(0.9987, 7.0622)	(0.9987, 7.0622)	0.0547	0.0547	0.0547
$17 \times 17$	8	(0.9990, 8.0545)	(0.9990, 8.0545)	(0.9990, 8.0545)	0.0420	0.0420	0.0420
$19 \times 19$	9	(0.9992, 9.0485)	(0.9992, 9.0485)	(0.9992, 9.0485)	0.0333	0.0333	0.0333
$21 \times 21$	10	(0.9994, 10.0436)	(0.9994, 10.0436)	(0.9994, 10.0436)	0.0270	0.0270	0.0270
$23 \times 23$	11	(0.9995, 11.0397)	(0.9995, 11.0397)	(0.9995, 11.0397)	0.0223	0.0223	0.0223

The superscript variables *n* and $\Omega$ reflect computations based on Farey sequences and size of chamfer masks, respectively.

### Efficient chamfer distance transforms

Depending upon the application, distance transforms can be engineered in two modes: parallel and sequential, with the later being popular in hardware implementations because of its regularity properties and implementation convenience. In sequential DTs, which the current research has adopted, the chamfer mask is divided into two halves, namely forward and backward masks, which operate antagonistically to generate chamfer polygons/balls (also called distance maps if operations are performed on binary images).

**Figure 2:**
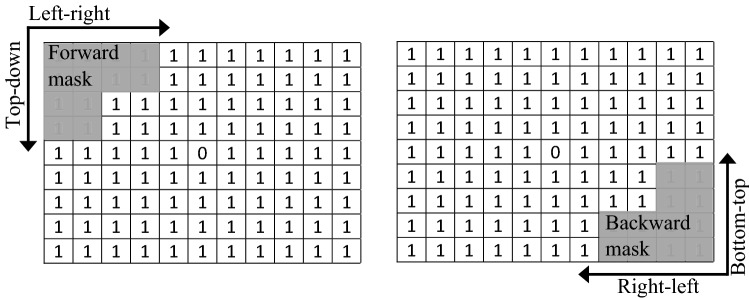
Operation of sequential distance transform.

Sequential DTs undertake two passes to compute chamfer balls. In the first pass, the forward mask slides in the left-right and top-down directions (Fig. 2). For every movement step, mask values and corresponding image pixel values are added to create a vector of distances. Next, the minimum distance in the vector is evaluated to replace a respective pixel just below the mask’s central value. This process continues until the mask perfectly covers pixels on the most bottom-right corner of the image. During the second pass, similar operations are repeated, but with the backward mask slided in the right-left and bottom-top directions.

The sequential chamfer distance transform requires time-consuming manual optimizations and configurations of the chamfer masks. These complexities increase exponentially with the dimensionality of the masks. Therefore, the available distance transform algorithms cannot satisfactorily support the time-sensitive industrial applications, especially those demanding high accuracy. One could, however, address the challenge through dedicated hardware with parallel computing capabilities. But this attempt calls for a huge financial investment, making the hardware unaffordable in resource-limited areas. Furthermore, previous approaches limit the process of generating optimal errors associated with the high-order chamfer masks.

We have, in this work, proposed an algorithm that uses only dimension of the mask as an input to compute distance transform of a binary image (Algorithm 1). In other words, given the desired size of the chamfer mask, we can automatically generate the distance map of the binary image. The function to achieve this goal accepts two inputs, namely binary image and chamfer mask dimension. Functions proposed by previous scholars require additional inputs, including coefficients of the chamfer masks that should analytically be computed.

Similar to the classical sequential algorithms, our algorithm contains forward and backward passes that operate sequentially to compute the distance maps. The algorithm embeds two external functions (Procedures 1 and 2): the first function traces the structure of the Farey sequence, and retrieves the number of terms, numerators, and denominators; and, the second function computes the values of the optimum chamfer weights. Procedure 2 shows that the process of optimizing the chamfer masks can be achieved with minimum computational load. The complexity of this Procedure is ${\mathcal {O}}(C)$, where *C* denotes a constant value that depends on the processor specifications. In essence, the Procedure needs only the size of the chamfer mask as input, and does not require iterations during optimization—hence making the process fast compared with other approaches.

### Experiments

Various experiments were executed to evaluate the efficacy of our approaches to generate optimum chamfer weights of arbitrary-shaped two-dimensional chamfer masks. The experiments, in addition, focused on computing distance maps of binary objects and on evaluating RLog errors associated with such maps. In the first experiment, the proposed algorithms were applied on chamfer masks of different sizes to generate RLog errors using either Farey sequences or dimensions of the masks. The second experiment was configured to generate RLog error lobes exhibited by low- and high-order chamfer masks. In the last experiment, we applied our algorithms to real-world binary images, and the objective of this experiment was to learn more on how the proposed approaches may suit practical applications, including those dwelling in computer vision and machine learning. In this last experiment, the distance maps generated by Euclidean, Chamfer metric, and our method were compared and evaluated to recognize a superior one.
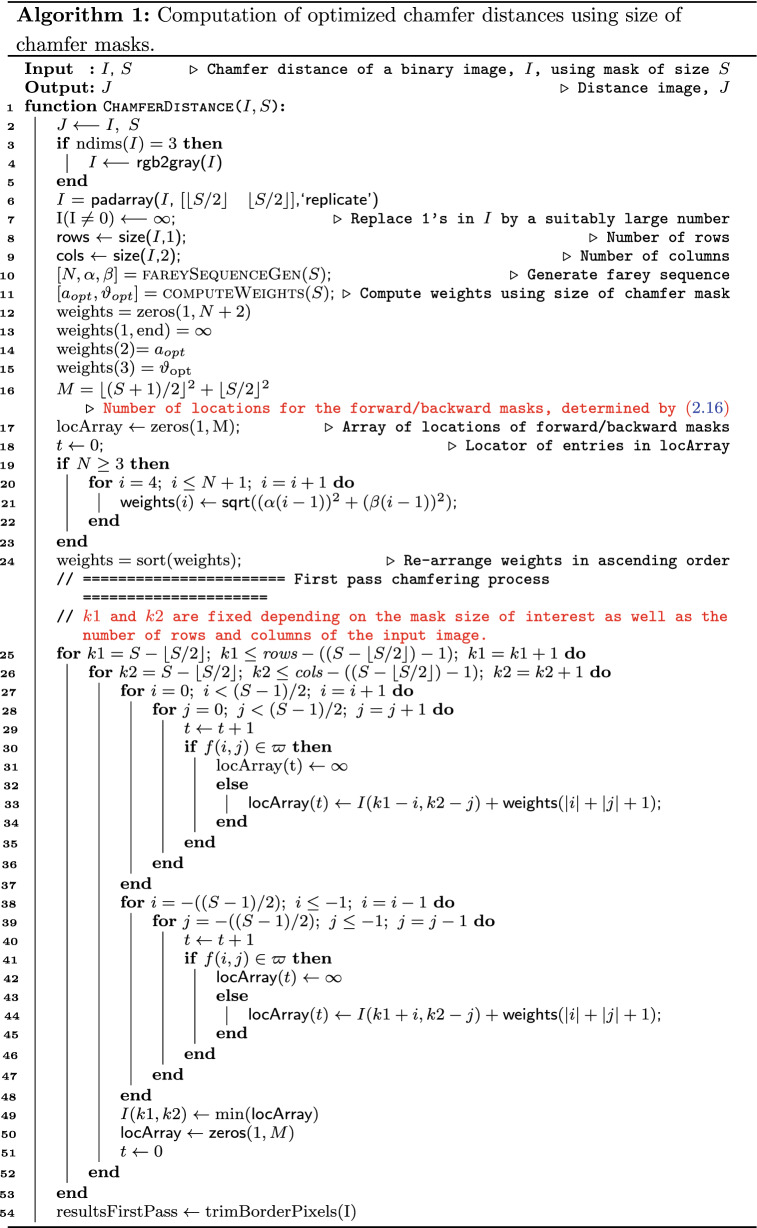

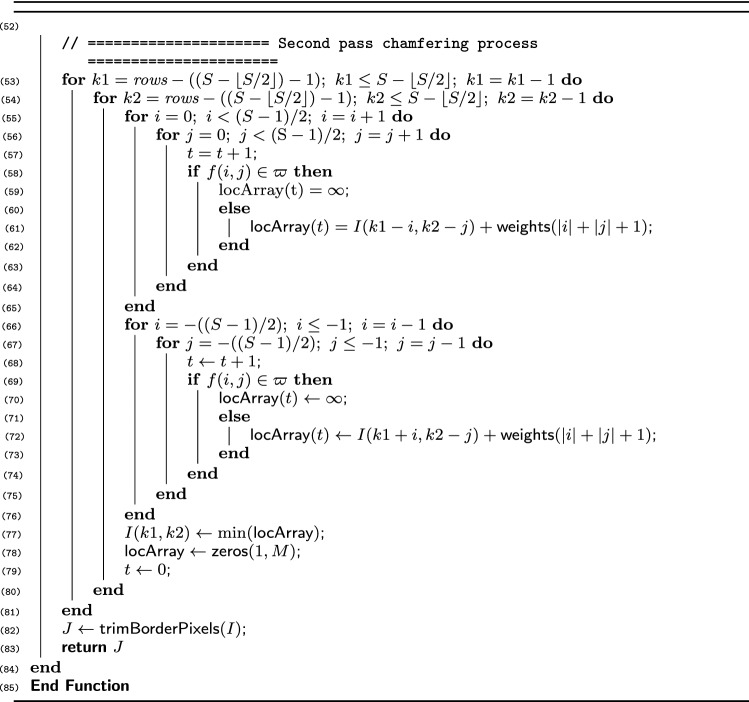

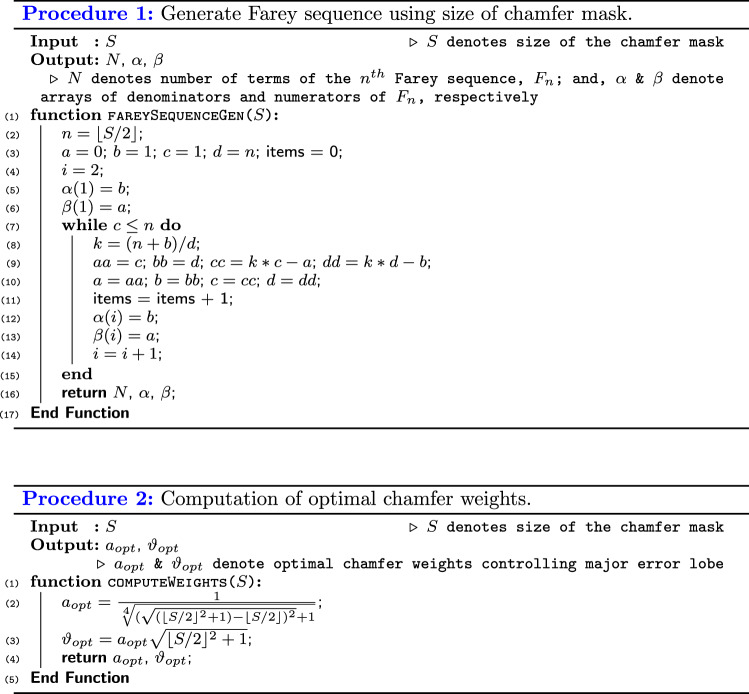


The experiments were conducted using MATLAB R2019a and laptop with the following specifications: RAM, 8 GB; Hard Disk, 1TB; Operating System, 64-bit Windows 10; and, processor, core i7 3.2 GHz speed. We ensured same experimental conditions, and the input parameters for all experiments were kept uniform. For instance, RLog errors depicted by the same shape of the chamfer mask were compared against different methods to establish a fairly simple comparison ground. To reproduce our results, implementation codes have been uploaded in the MATLAB File Exchange Central (https://www.mathworks.com/matlabcentral/fileexchange/71652-optimization-of-chamfer-masks).

## Results and discussions

We found that Farey sequences can effectively be used to optimize chamfer masks. Furthermore, the current study revealed that shape (dimension) of the chamfer mask can be applied to introduce convenience when generating more accurate distance maps from high-order chamfer masks. Figure 4 shows that error lobes of larger masks above $105\times 105$ can be achieved through simple formulations that use dimensionality of the chamfer masks. In the work by Butt and Maragos^17^, error lobes associated with $21 \times 21$ chamfer masks could be displayed. Although the authors’ concepts could be extended to larger masks, this extension could be achieved at the expense of increased complexity in their formulations.

Figures 5 and 6 show the distance maps of electronic components. For performance evaluation, the comparison is usually done with respect to the distance maps generated by the (reference) Euclidean metric. The visual results from Figs. 5 and 6 demonstrate that our distance metric generates maps with minimal errors, and which are comparable with those generated by the Euclidean and chamfer metrics.

Previous scholars have never exploited structure of the chamfer masks to devise effective approaches to compute optimum weights of the chamfer masks. Therefore, our attempts provide some critical research insights on how we can establish faster algorithms for generating distance maps (Figs. 5, 6). Furthermore, practitioners may embed such effective algorithms into dedicated hardware that can be deployed into the real environment. Our algorithm to compute distance maps has convenience as a major advantage: the algorithm accepts a single input argument, dimension of the chamfer mask, and generates relatively more accurate distance maps of a binary image. Therefore, a scholar needs only to select the chamfer mask’s dimension for the desired distance map. One should note that the proposed algorithm uses this (dimension of the chamfer mask) argument to implicitly compute the optimum weights of the chamfer masks, which are necessary to compute the distance maps.

The current research has, in addition, demonstrated another application of Farey sequences in designing chamfer masks. We have established elegant relationships between such sequences and weights of the chamfer masks. This contribution sets another perspective of Farey sequences in computer vision and image processing, and scholars may attempt to extend the established relationships to three-dimensional chamfer masks. This further extension may allow the algorithm to be applied in other sensitive fields, including medical imaging where three-dimensional images of body organs are common^22,23^.

In this work, we have provided formulations and derivations that may be suitable to advance the field of chamfer metrics. In Refs.^20,21^, the author gave an error metric, called symmetric mean absolute percentage error (SMAPE), for selecting more accurate weather forecasting models. This metric, defined by

18 $$\begin{aligned} Y(\theta )=\frac{|r-f(\theta )|}{r+f(\theta )}, \end{aligned}$$

may be used to generate optimal chamfer weights similar to those generated by RLog. The variables on the right side of (18) are defined in Fig. 1. Using Farey sequences and shapes of chamfer masks, one can intuitively show that the respective maximum values of *Y* are

19 $$\begin{aligned} Y(n)=\frac{\sqrt{\left( \sqrt{n^2+1}-n\right) ^2+1}-\root 4 \of {\left( \sqrt{n^2+1}-n\right) ^2+1}}{\sqrt{\left( \sqrt{n^2+1}-n\right) ^2+1}+\root 4 \of {\left( \sqrt{n^2+1}-n\right) ^2+1}}, \end{aligned}$$

and

20 $$\begin{aligned} Y(\Omega )=\frac{\sqrt{\left( \sqrt{\left\lfloor \frac{\Omega }{2}\right\rfloor ^2+1}-\left\lfloor \frac{\Omega }{2}\right\rfloor \right) ^2+1}-\root 4 \of {\left( \sqrt{\left\lfloor \frac{\Omega }{2}\right\rfloor ^2+1}-\left\lfloor \frac{\Omega }{2}\right\rfloor \right) ^2+1}}{\sqrt{\left( \sqrt{\left\lfloor \frac{\Omega }{2}\right\rfloor ^2+1}-\left\lfloor \frac{\Omega }{2}\right\rfloor \right) ^2+1}+\root 4 \of {\left( \sqrt{\left\lfloor \frac{\Omega }{2}\right\rfloor ^2+1}-\left\lfloor \frac{\Omega }{2}\right\rfloor \right) ^2+1}}. \end{aligned}$$

In essence, Farey sequences become useful in situations where larger chamfer masks are needed to compute more accurate distance transforms in binary images. Because of the relationship between Farey sequences and size of the chamfer masks, we have demonstrated that optimal weights of large-size chamfer masks can easily be computed using closed-form equations, an advantage that has never been realized before. Our belief is that the discovery made in this work may open new research directions in image processing and computer vision.

**Figure 3:**
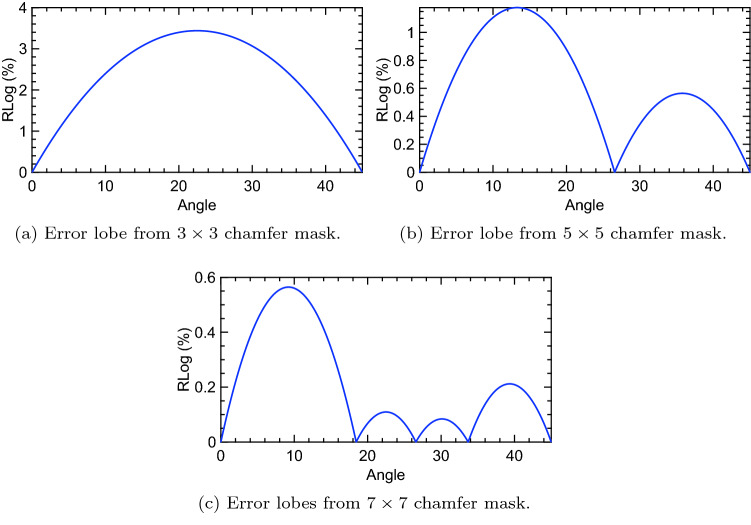
RLog error lobes generated by low-order chamfer masks.

**Figure 4:**
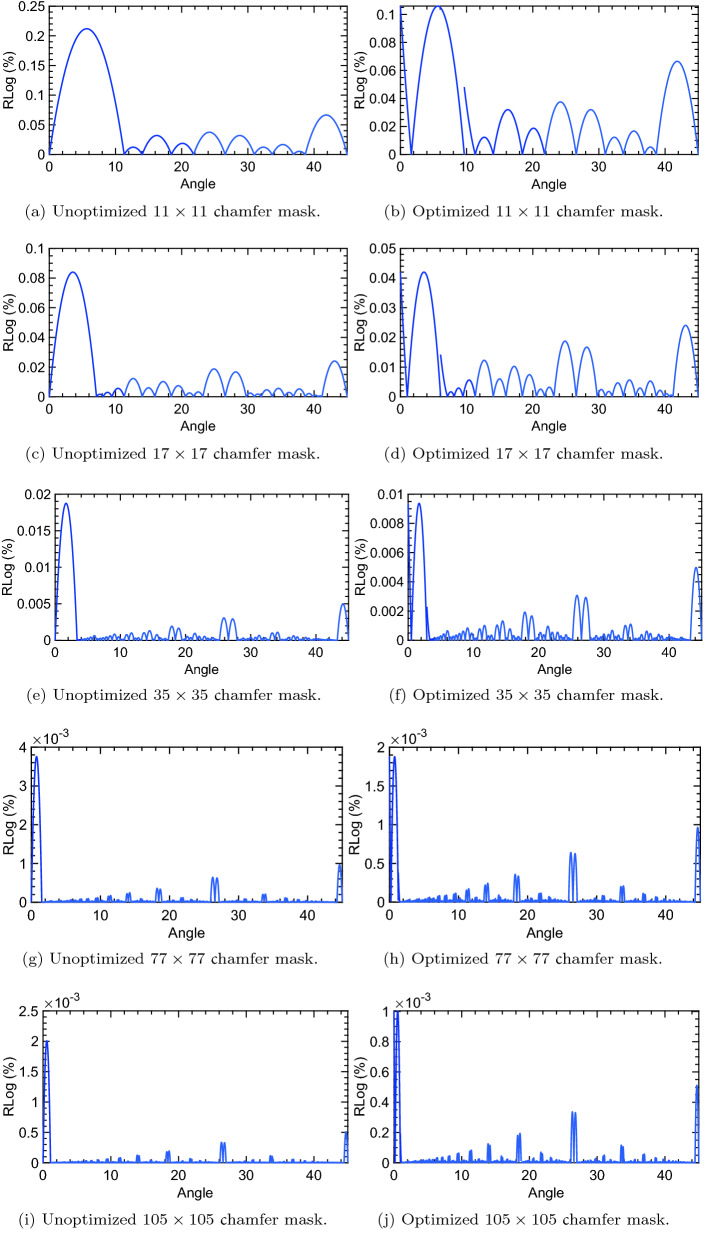
RLog Error lobes generated by high-order chamfer masks.

**Figure 5:**
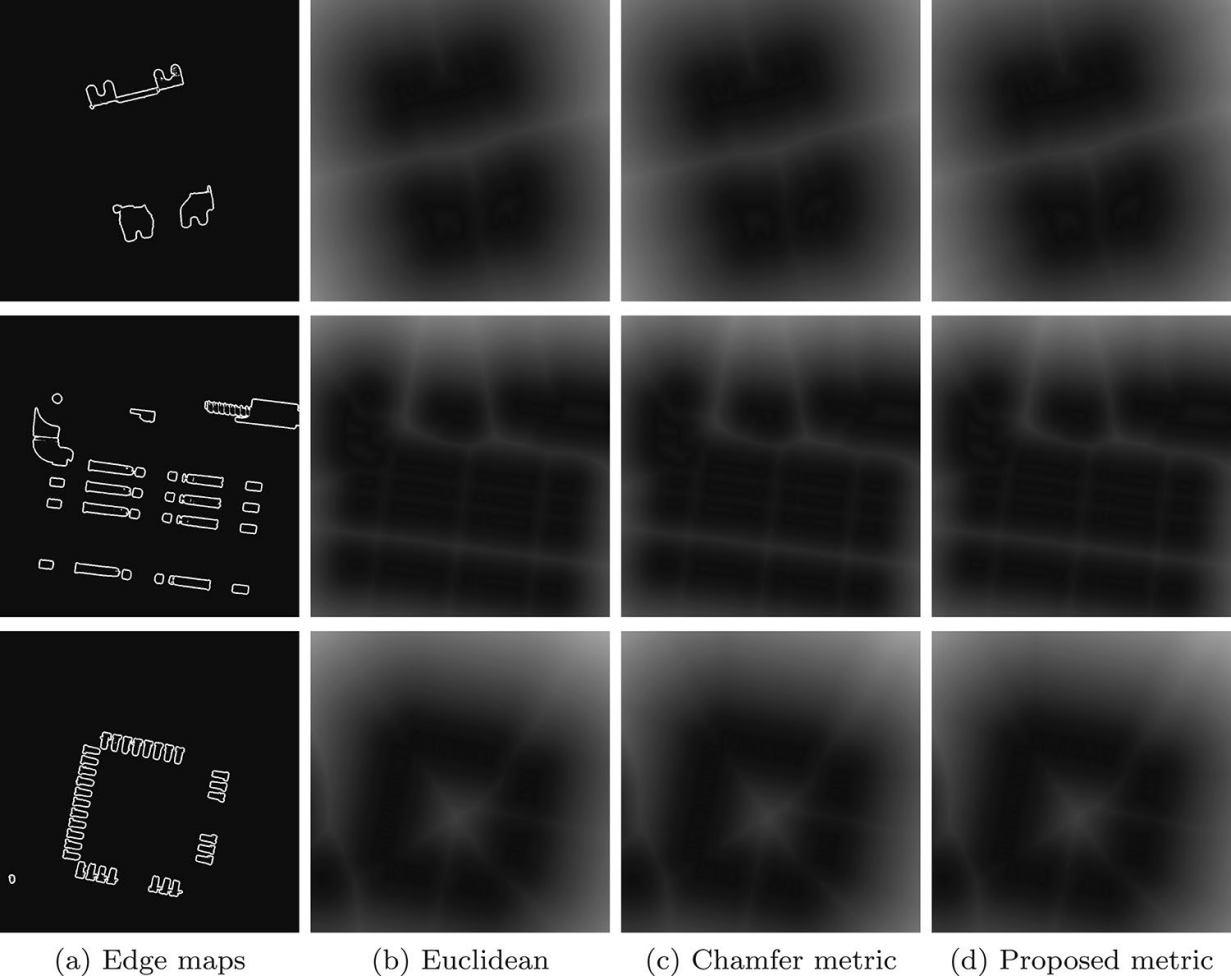
Distance maps generated by various metrics applied on real binary images.

**Figure 6:**
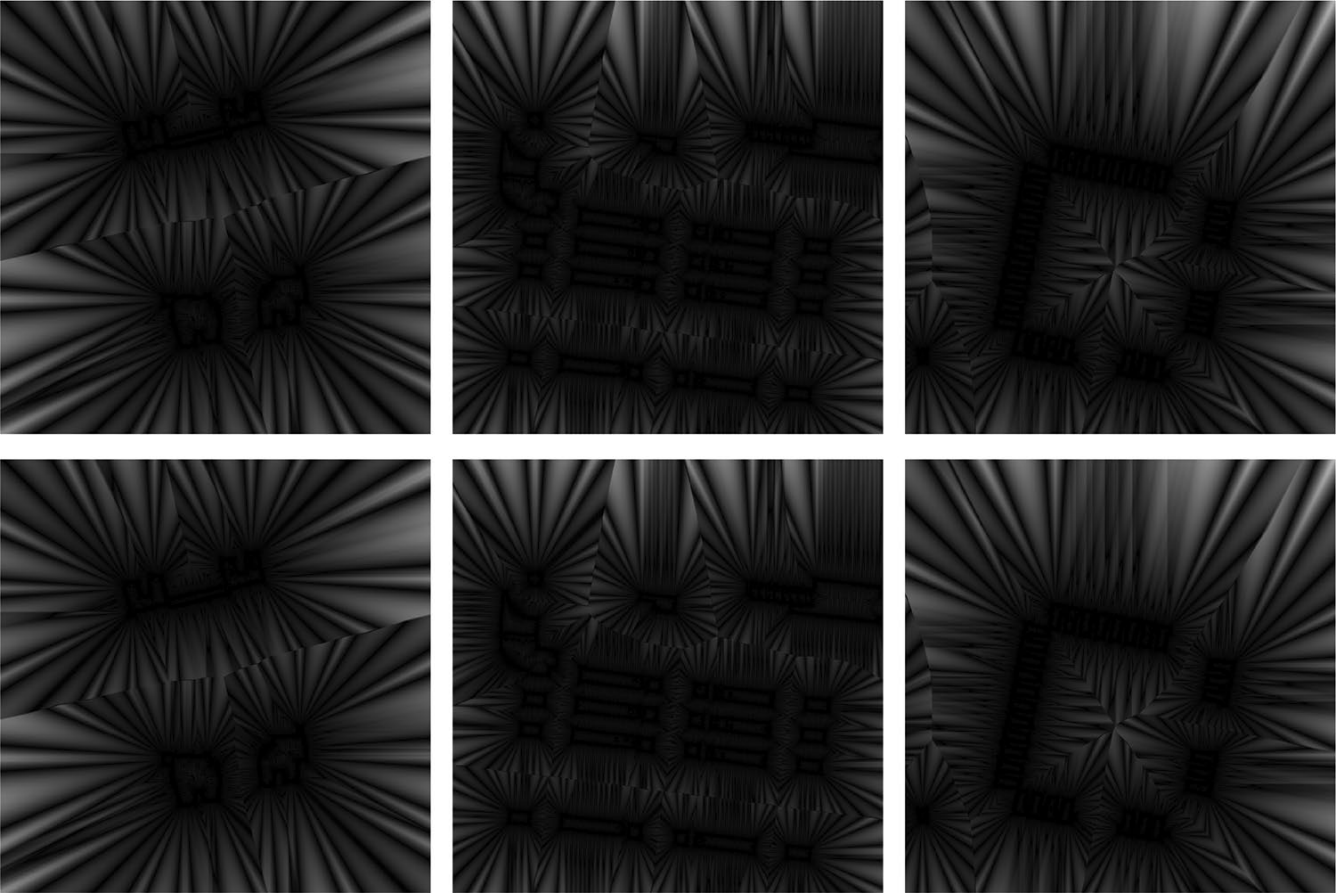
Error maps generated by different metrics: first row, Butt and Maragos; and, second row, our approach.

## Conclusion

The knowledge on Farey sequences has never been extended and applied to optimize chamfer masks. In this work, we have demonstrated that such sequences are important, and can provide simple closed-form equations to compute optimal local weights of chamfer masks. In addition, the work gives mathematical relationships between shapes of chamfer masks and optimal chamfer weights. Compared with the earlier work by Maiseli et al., “Robust cost function for optimizing chamfer masks”, we have demonstrated that the same results can be obtained with the proposed equations that are simpler and effective. Finally, we have established algorithms with minimum number of input arguments to more effectively compute error maps associated with arbitrary-shaped chamfer masks. The algorithms can work well in the optimization of even high-order chamfer masks. In future, researchers may consider expanding our methods and algorithms to deal with chamfer masks suitable for computing distance maps of multi-dimensional objects, commonly found in the field of medical imaging. Also, it may be interesting to further explore the applications of our formulations in other fields.

Farey sequences serve as an important tool to efficiently optimize chamfer masks. This advantage may be useful in the implementation of embedded systems for computing distance transforms of complex digital images. Results from our work show that Farey sequences can be used to optimize chamfer masks based on their dimensionality. Despite these advantages, researchers may attempt to extend our results to three-dimensional and high-order chamfer masks. Subsequently, distance transform algorithms based on Farey sequences and dimensionality of chamfer masks are needed.

## Data Availability

The link with supporting data and implementation codes has been included in this paper.

## Abbreviations

DT: Distance transform
RLog: Relative logarithm
